# Prevalence, intensity and extent of Oral Impacts on Daily Performances associated with self-perceived malocclusion in 11-12-year-old children

**DOI:** 10.1186/1472-6831-7-6

**Published:** 2007-05-16

**Authors:** Eduardo Bernabé, Carlos Flores-Mir, Aubrey Sheiham

**Affiliations:** 1 Unidad de Investigación en Salud Pública Dental, Departamento de Odontología Social, Universidad Peruana Cayetano Heredia, Lima, Perú; 2 Department of Epidemiology and Public Health, University College London, London, UK; 3 Department of Dentistry and Cranio-facial & Oral-Health Evidence-based Practice Group, Dentistry/Pharmacy Centre, University of Alberta, Edmonton, Canada

## Abstract

**Background:**

To determine the prevalence, intensity and extent of the Oral Impacts on Daily Performances associated with self-perceived malocclusion among Peruvian schoolchildren.

**Methods:**

Eight hundred and five children aged 11 to 12 years attending 4 of 7 randomly selected schools linked to a Health Centre in Lima, Peru, participated in the study. The Spanish _(Peru)_Child-OIDP was used to assess the prevalence, intensity and extent of oral impacts on 8 daily performances (eating, speaking, teeth cleaning, sleeping, smiling, studying, emotion and social contact). Self-perceived malocclusion included complaints about position of teeth, spacing of teeth and deformity of mouth or face. The prevalence of oral impacts was compared by covariables using the Chi-square test, whereas the intensity and extent of oral impacts were compared by covariables through the Mann-Whitney test.

**Results:**

Only 15.5% of children reported impacts associated with self-perceived malocclusion during the last 3 months. Of them, 18.4% reported impacts of severe or very severe intensity and 76.0% reported impacts on only one daily performance. Psychosocial activities such as smiling, emotion and social contact were the most frequently and severely impacted everyday activities.

**Conclusion:**

Impacts of self-perceived malocclusion primarily affected psychological and social everyday activities. These findings provide further evidence to support the importance of psychological and social components of oral health on children's lives.

## Background

A better knowledge about the physical, social and psychological effects of malocclusion is important since it provides insights into the perceived impacts of malocclusion on children's lives [1,2]. To date, there is conflicting evidence on the impact of malocclusion on quality of life. A recent review concluded that a greater understanding is required of the physical, psychological and social consequences of malocclusion [3]. Therefore, there is a need for a more comprehensive and rigorous assessments of the impacts of malocclusion on quality of life. These assessments should be done on representative population-based epidemiological samples, rather than using patient-based studies, and through the use of specific, rather than generic, oral health related quality of life (OHRQoL) measures.

Specific OHRQoL measures are designed for use in clinical situations. Their narrow focus means that they are potentially more responsive to small, but clinically important, changes in health [2,4,5]. Specific instruments may be divided into four types: (1) condition-specific; focusing on individuals with a particular disease or clinical condition, (2) domain-specific; focusing in detail on one dimension only, such as psychological domain, (3) population-specific; focusing on subgroups of people such as elderly or children, and (4) symptom-specific; focusing on one type of symptom, such as pain [4,6].

Condition-specific instruments are the most commonly used specific measures to assess quality of life [2]. The advantage of these instruments is that the emphasis is on a specific area of quality of life, rather than assessing quality of life globally [5,6]. Although some OHRQoL measures have been specifically developed to assess the impact of oral characteristics on children's day-to-day living [7-9], the child version of the Oral Impacts on Daily Performances (Child-OIDP) is the only one specifically designed to identify the oral problems leading to the impacts on quality of life, thereby linking the impacts to the oral condition needing attention [9]. To make the link, participants are asked about oral problems they consider cause the impacts on their daily living. The condition-specific Child-OIDP (CS-Child-OIDP), based on the calculations related to specific oral conditions, can assist in deciding which groups of children should be treated first [10,11]. This characteristic has enabled its use in the assessment of treatment needs [12,13].

Previous studies assessing the impact of malocclusion on children or adolescents have only reported on the prevalence of impacts on quality of life [14-16]. No study has explored the intensity or the extent (number of affected daily activities) of the impacts associated with perceived malocclusions. Therefore, the objective of this study was to determine the prevalence, intensity and extent of the oral impacts associated with self-perceived malocclusion among Peruvian schoolchildren.

## Methods

The study was carried out within the area related to the Mother-Child Health Centre of Zapallal in Puente Piedra (Lima, Peru). There are 7 public schools in this jurisdiction. In 2006, 1519 children aged 11 to 12 years (born in 1995 and 1994 respectively) attended these schools. 4 of these 7 schools were randomly selected as clusters, and all their 903 11-12-year-old children were invited to participate in the study. Ethical approval was obtained from the International Review Board at the Universidad Peruana Cayetano Heredia. Parents signed a consent letter accepting participation of their children. Children also gave written consent for interviews.

Before data collection, the original English version of the Child-OIDP was cross-culturally translated and adapted into Spanish. Then, the validity and reliability of the Spanish _(Peru)_Child-OIDP were evaluated. For criterion validity, the Spanish _(Peru)_Child-OIDP scores were significantly associated with self-perceived oral health status, self-perceived dental treatment need and satisfaction with oral health status (p < 0.001 in all cases). For internal reliability, all inter-item correlations were positive and statistically different from zero (p ≤ 0.007), whereas the Cronbach's alpha coefficient was 0.62 and did not increase when any performance was deleted. Finally, test-retest reliability was evaluated through intraclass correlation coefficient, whose value was 0.85.

The Child-OIDP was administered through individual interviews, except for the first question that was self-administered in a classroom setting. The children were first asked to provide socio-demographic information related to their sex, age and education level (e.g. primary or secondary school), and then, to identify problems with their mouth or teeth perceived during the last 3 months. Thereafter, 2 trained interviewers carried out individual face-to-face structured interviews. Oral impacts on daily life were assessed in relation to 8 daily performances namely, eating, speaking, mouth cleaning, sleeping, smiling, studying, emotion and social contact. If children reported an impact on any performance, the frequency of the impact (scale from 1 to 3) and the severity of its effect on daily life (scale from 1 to 3) were scored [9]. If no impact was reported, then a zero score was assigned. Finally, children with impacts were asked to identify the oral conditions they perceived as causes of their impacts, using the list of answers to the self-administered question. Although children reported a number of oral problems as causes of their impacts, for this study only those associated with 'bad position of teeth', 'spacing of teeth' and/or 'deformity of mouth or face' were analysed to calculate the condition-specific impacts for malocclusion, hereafter referred to as the CS-Child-OIDP. Because no normative data were collected in this study, the information represents impacts related to self-perceived malocclusion.

The impact per daily performance was estimated by multiplying the corresponding frequency and severity scores. The overall CS-Child-OIDP score was the sum of the 8 performance scores (ranging from 0 to 72) multiplied by 100 and divided by 72 [9]. Then, the prevalence of oral impacts on daily performances was calculated as the percentage of children with a CS-Child-OIDP score higher than zero. Furthermore, among those children reporting oral impacts, the intensity of the impact on each performance score (ranging from 1 to 9) was classified into 5 levels: very little (1), little (2), moderate (3–4), severe (6) and very severe (9) [17,18]. The overall intensity of impacts was then estimated as the most severe impact on any of the 8 performances [17]. Finally, the extent of impacts, ranging from 1 to 8 performances, was calculated as the number of performances with impacts [17,18].

For the statistical data analysis, the prevalence of condition-specific impacts was compared according to sex, age and education level using the Chi-square test. The intensity and extent of condition-specific impacts were compared according to covariables using the Mann-Whitney test. Non-parametric tests were used to compare intensity and extent because the former was measured using an ordinal scale and the latter was not normally distributed in the sample (Kolmogorov-Smirnov test, p < 0.05 for all groups).

## Results

Eight hundred and five of the 903 11–12 year-old children attending the 4 selected schools participated in the study; a response rate of 89.1%. Fifty one percent (51.2%) were female and 48.8% male; 53.5% were at primary education and 46.5% at secondary education level. The mean age of the children was 11.93 ± 0.63 years.

The prevalence of self-perceived malocclusion was 36.3% [CI_95%_(32.9; 39.6)]. The most frequently reported oral condition related to malocclusion was position of teeth (28.4%), followed by spacing of teeth (16.3%) and deformity of mouth or face (1.6%). The prevalence of self-perceived malocclusion did not differ by sex or age (p = 0.275 and 0.057 respectively). However, there was a significant difference of perceived malocclusion by education level (p = 0.021) (Table 1).

**Table 1:** Prevalence of self-perceived malocclusion among 11-12-year-old Peruvian schoolchildren

**Covariables**	**Perceived malocclusion**		**No perceived malocclusion**		**p value**
		
	**n**	**%**	**n**	**%**	
*Sex*					0.275
Girls	142	34.5	270	65.5	
Boys	150	38.2	243	61.8	
*Age*					0.057
11 years	91	41.6	128	58.4	
12 years	201	34.3	385	65.7	
*Education level*					0.021
Primary school	172	39.9	259	60.1	
High school	120	32.1	254	67.9	

The prevalence of condition-specific impacts was 15.5% [CI_95%_(13.0; 18.1)]. The most common daily performances affected by malocclusion were smiling and emotion (9.1% and 3.2% respectively). The prevalence of condition-specific impacts on the other evaluated 6 daily performances was less than 2.0% (Table 2). There was no statistically significant difference when the prevalence of condition-specific impacts was compared by covariables (p > 0.118 in all cases) (Table 3).

**Table 2:** Prevalence and intensity of the impacts associated with self-perceived malocclusion among 11-12-year-old Peruvian schoolchildren

**Indicator**	**Impact by daily performances**								**Overall impact**
		
	**Eating**	**Speaking**	**Cleaning mouth**	**Sleeping**	**Emotion**	**Smiling**	**Studying**	**Social contact**	
*Prevalence of impacts (n = 805 children)*									
n	10	13	15	6	26	73	5	15	125
%	1.2	1.6	1.9	0.7	3.2	9.1	0.6	1.9	15.5
*Intensity of impacts (children with impacts)*									
Very little	40.0	46.2	20.0	50.0	26.9	28.8	40.0	20.0	28.8
Little	60.0	38.5	53.3	50.0	38.5	23.3	40.0	33.3	34.4
Moderate	0.0	7.7	20.0	0.0	23.1	19.2	20.0	20.0	18.4
Severe	0.0	7.7	6.7	0.0	7.7	20.5	0.0	20.0	13.6
Very severe	0.0	0.0	0.0	0.0	3.8	8.2	0.0	6.7	4.8

**Table 3:** Prevalence of the impacts associated with self-perceived malocclusion, by covariables, among 11-12-year-old Peruvian schoolchildren

**Covariables**	**Impacts**		**No impacts**		**p value**
		
	**n**	**%**	**n**	**%**	
Sex					0.118
Girls	72	17.5	340	82.5	
Boys	53	13.5	340	86.5	
*Age*					0.828
11 years	35	16.0	184	84.0	
12 years	90	15.4	496	84.6	
*Education level*					0.443
Primary school	63	14.6	368	85.4	
High school	62	16.6	312	83.4	

Mann- Whitney test was used

Among children with condition-specific impacts, 18.4% [CI_95%_(11.5; 25.3)] reported impacts of severe or very severe intensity (Table 2). In the analysis by performances, smiling and social contact were the most severely impacted daily performances; 28.7% and 26.7% of the children with condition-specific impacts reported impacts of severe or very severe intensity respectively, whereas eating, sleeping and studying were the least severely impacted performances and no child reported condition-specific impacts of severe or very severe intensity for them. The intensity of condition-specific impacts differed only between education levels (p = 0.029) (Table 4).

**Table 4:** Intensity of the impacts associated with self-perceived malocclusion, by covariables, among 11-12-year-old Peruvian schoolchildren

**Covariables**	**Intensity of the impacts**										**p value**
		
	**Very little**		**Little**		**Moderate**		**Severe**		**Very severe**		
		
	**n**	**%**	**n**	**%**	**n**	**%**	**n**	**%**	**n**	**%**	
*Sex*											0.187
Girls	14	19.4	32	44.4	13	18.1	9	12.5	4	5.6	
Boys	22	41.4	11	20.8	10	18.9	8	15.1	2	3.8	
*Age*											0.447
11 years	13	37.2	10	28.6	6	17.1	4	11.4	2	5.7	
12 years	23	25.6	33	36.7	17	18.9	13	14.4	4	4.4	
*Education level*											0.029
Primary school	23	36.6	22	34.9	9	14.2	7	11.1	2	3.2	
High school	13	21.0	21	33.8	14	22.6	10	16.1	4	6.5	

Mann- Whitney test was used

The mean number of performances impacted was 1.30 [IC_95%_(1.19; 1.41)], with 76.0% of children with condition specific impacts reporting 1; 19.2% reporting 2, 3.2% reporting 3, 0.8% reporting 4 and 0.8% reporting 5 performances affected. None reported condition-specific impacts on 6 or more of the 8 daily performances. No statistically significant difference was found when the extent of the condition-specific impacts was compared by covariables (p > 0.344 in all cases) (Table 5).

**Table 5:** Extent of the impacts associated with self-perceived malocclusion, by covariables, among 11-12-year-old Peruvian schoolchildren

**Covariables**	**n**	**Performances with impacts**				**p value**
			
		**Mean**	**S.D.**	**Range**	**Quartiles**	
*Sex*						0.725
Girls	72	1.32	0.71	1 – 5	(1, 1, 1)	
Boys	53	1.28	0.50	1 – 3	(1, 1, 2)	
*Age*						0.883
11 years	35	1.29	0.57	1 – 3	(1, 1, 1)	
12 years	90	1.31	0.65	1 – 5	(1, 1, 1.25)	
*Education level*						0.344
Primary school	63	1.24	0.50	1 – 3	(1, 1, 1)	
High school	62	1.37	0.73	1 – 5	(1, 1, 2)	

Mann- Whitney test was used

## Discussion

The present study is the first to assess the intensity and extent of the impacts associated with perceived malocclusion on the quality of life of children. We used self-perceived malocclusion rather than a normative definition of malocclusion since normative orthodontic need indexes are not strongly associated with people's perceptions of their oral health status [19,20] and quality of life [3,14,15]. Subjective impacts directly identified as being related to malocclusion have been previously used to capture subjective feelings [21,22]. Although a similar group of oral conditions has been used in the development of a socio-dental model to assess children's orthodontic needs [12], it should be noted that impacts associated to spacing of teeth among 11–12 year-old children may include spaces due to unerupted permanent teeth or physiological diastemas rather than spaces indicating a need for orthodontic treatment. A proper discrimination would only be possible through a comprehensive clinical examination, which was not done in this study.

Our results indicated that 36.6% of the children reported self-perceived malocclusion. Of the three conditions associated by the children with malocclusion, position of teeth was the most, and mouth and face deformity, the least frequent. Similar findings were reported among Thai [17] and French [23] children. However, only 42.8% of the children with self-perceived malocclusion (15.5% of the whole sample) reported impacts on at least one of the 8 daily performances during the last 3 months. That figure was similar to that reported in the only previous population-based study carried out among 11-12-year-old Thai children (20.3%) [12]. At present, orthodontic care in Peru is only provided by the fee-for-service modality, which makes it expensive and unaffordable for most people [19,24]. Since orthodontic treatment is not offered in public health services, different expectations about malocclusion and its treatment can be expected. Different norms for acceptable dental arrangement operate in areas with low and high treatment frequency [25].

Slightly less than one-fifth of the Peruvian children with condition-specific impacts reported severe or very severe intensity. Children reported a greater prevalence and intensity of impacts related to smiling, laughing and showing the teeth without embarrassment, contact with people (e.g. going out with friends, going to a friend's house) and maintaining the usual emotional state without being irritable. Other psychosocial activities such as carrying out schoolwork (e.g. going to school, participating in class, doing homework) and relaxing/sleeping (e.g. watching television, reading a comic book) were the least frequent and least severely impacted. These findings highlight the importance of the psychological and social aspects of the teeth and mouth on children's lives. Teeth mainly affect social interaction with peers, where satisfaction with dental appearance plays a very important role [16,20,26].

Interference with predominantly physical activities such as eating and enjoying food, speaking clearly and cleaning mouth (e.g. rinsing your mouth, brushing your teeth) were only reported by between 1.3% and 1.9% of the children. It has been reported that dissatisfaction with ability to chew was less often a reason for seeking orthodontic treatment because problems with chewing may be less common than problems related with dental appearance [15,27]. These findings question long-standing beliefs that the main effects of malocclusion are on mastication and speech. However, it is difficult to draw firm conclusions about these issues from this study.

More than three-quarters of the children with impacts had only one daily performance affected. The performances affected were mainly related to smiling, laughing and showing teeth without embarrassment. Eating, sleeping and studying were not frequently affected. No child reported impacts on more than 5 daily performances. The findings support the view that children with a perceived malocclusion are more concerned with dental aesthetics than with function. Therefore, psychological factors, such as dental aesthetics, self-perception of dental appearance, rather than the severity of the clinical occlusal condition, most probably determine children's demand for orthodontic treatment [27,28].

The Child-OIDP measures impacts on the ultimate level of the oral impacts [9,29], equivalent to the disability and handicap dimensions in the WHO model [30]. Measuring impacts only at the ultimate level covers all major impacts, and omits very minor intermediate level conditions thereby avoiding over-scoring when intermediate impacts are also measured [31].

If the figures reported here were used for orthodontic services planning, the estimates of orthodontic treatment need would likely be lower than those obtained using normative indexes alone [12,14,15]. Using the prevalence and intensity of oral impacts associated with perceived malocclusion, only 125 (15.5%) and 23 (2.9%) respectively of the 805 children would require orthodontic treatment based on their subjective perceptions. Although normative needs and OHRQoL are associated, there is a considerable difference between them. Therefore, OHRQoL measures cannot replace normative needs or vice-versa [32]. Instead, both should be used in combination to cover different dimensions of oral health. Since the normative approach to estimate orthodontic needs gives unrealistically high estimates of need, a more realistic method of assessing needs requires the integration of a normative measure with an indicator of the child's feelings and/or oral impacts [12,13]. Using such a socio-dental model to assess orthodontic need is more appropriate for dental service planning. It provides more appropriate manpower estimates based on the potential for oral health gain [10,11].

Although it has been argued that the physical, social and psychological effects are important reasons why orthodontic care is sought [3], our findings support the idea that the impact of the malocclusion, at least the self-perceived, primarily affected the psychological and social components of oral health and those factors may induce demand. However, a recent 20-year longitudinal study concluded that there was little objective evidence to support the assumption that orthodontics improves long-term psychological health. Thus, orthodontics cannot be justified on psychological grounds alone [33]. Therefore, further studies are needed to provide a greater understanding of the consequences of malocclusion, the effects of malocclusion if left untreated, and also the possible benefits of orthodontic care on day-to-day activities. These studies should be based not only on normative need but also on OHRQoL information acquired from the children. That would improve orthodontic treatment need assessments [12].

## Conclusion

- Only 15.5% of the interviewed children reported impacts associated with self-perceived malocclusion during the last three months. Among children with impacts, 18.4% reported impacts of severe or very severe intensity and 76.0% had impacts on only one daily performance.

- Among children with impacts, psychosocial activities such as smiling, emotion and social contact were the most frequently and severely impacted. These findings provide further evidence in support of the importance of psychological and social components of oral health children's lives.

- Education level was the only demographic variable that significantly affected the prevalence of self-perceived malocclusion and intensity of the impacts associated with self-perceived malocclusion.

## Competing interests

The author(s) declare that they have no competing interests.

## Authors' contributions

Eduardo Bernabé conceived of the study, was responsible for the data collection in Peru, conducted the statistical analysis and was responsible for the completion of the whole manuscript. Carlos Flores-Mir was consulted for methodology and revised the manuscript. Aubrey Sheiham contributed to the conception and design of the study and critically revised the manuscript. All authors read and approved the final version of the manuscript.

## Pre-publication history

The pre-publication history for this paper can be accessed here:


